# Supplementary material for: Development of a computer-aided design software for the quantitative evaluation of aesthetic damage
Source: PLoS One. 2019 Dec 18;14(12):e0226322. doi: 10.1371/journal.pone.0226322 (PMC6919621; doi:10.1371/journal.pone.0226322)
Supplement: S1 Code site — 1. (ZIP) [file pone.0226322.s001.zip › [EESC_jr][Projeto]Relatorio_07_21/vendors/select2/docs/_includes/options/compatibility/introduction.html]

Select2 offers limited backwards compatibility with the previously 3.5.x
release line, allowing people more efficiently transfer across releases
and get the latest features. For many of the larger changes, such as the
change in how custom data adapters work, compatibility modules were
created that will be used to assist in the upgrade process. It is not
recommended to rely on these compatibility modules, as they will not
always exist, but they make upgrading easier for major changes.

**The compatibility modules are only included in the
full builds of
Select2**. These files end in `.full.js`, and the
compatibility modules are prefixed with `select2/compat`.
